# PROTOCOL: Correlates and Antecedents of Hate Crime: A Systematic Review of Place‐Level Risk and Protective Factors

**DOI:** 10.1002/cl2.70048

**Published:** 2025-07-07

**Authors:** Kathryn Benier, Michelle Sydes, Angela Higginson

**Affiliations:** ^1^ Monash University Melbourne Victoria Australia; ^2^ Griffith University Brisbane Queensland Australia; ^3^ Queensland University of Technology – School of Justice Brisbane Queensland Australia

**Keywords:** bias crime, diversity, hate crime, neighbourhood, spatial, systematic review

## Abstract

This is a protocol for a Campbell Systematic Review. The review aims to synthesise the current empirical evidence on the places factors associated with hate crime victimisation. The review will also consider whether the risk and protective factors for hate crime vary according to moderating factors, such as the features of the crime, the victim, the place and cultural contextual factors.

## 1 Background

### 1.1 The Problem

Hate crimes – crimes motivated by prejudice, racism, intolerance or xenophobia – involve words or actions intended to harm or intimidate individuals because of their perceived membership in, or association with, another group (Craig and Waldo 1996). Evidence from the UK shows that over 70% of minority group members have been victimised in a hate incident (Paterson et al. 2018), with victims often targeted because of their race, ethnicity, religion, gender, sexual orientation or disability. While there is some debate in the literature about the groups to be covered by hate crime legislation, this review will include hate crimes that are motivated by race, religion, ethnicity, disability, gender identity and sexuality. Hate crimes are known to have detrimental impacts on the individual victim, as well as other group members who share the same minority group status as the victim (Benier 2017a; Boeckmann and Turpin‐Petrosino 2002; Perry 2002). Hate crimes also create divisive fractures for social cohesion within a neighbourhood (Perry and Alvi 2012).

Hate crime is a mechanism of power (Perry 2002). In this way, hate crime has the effect of establishing and enforcing a social hierarchy that places the victims and their minority group in a subordinate class, and continually reaffirms this social order (Weisburd and Levin 1993). Acts motivated by hate are not an individual‐based assault – they have been labelled a ‘message crime’ designed to target all members of stigmatised and excluded community groups by enhancing their sense of vulnerability and fearfulness (Perry 2002; Perry and Alvi 2012). Thus, hate crime directly threatens equality and social harmony by heightening divisions in society and undermining stability.

Antecedents of hate crime operate at both the individual and neighbourhood level, and there is debate in the literature about whether hate offences have unique drivers from other types of crime as they are driven by ‘hate’, or whether the underlying factors of the crime are similar to non‐hate offences (Benier 2019). This systematic review aims to synthesise the empirical evidence on the risk and protective factors for hate crimes that occur within a place‐based context. Studies that examine the place‐based correlates of hate crime level typically consider economic or financial strain, threats to community identity, and/or social disorganisation or routine activity theories (see, e.g., Benier et al. 2016; Gladfelter et al. 2015; Green et al. 1998; Lyons 2008; Van Kesteren 2016). Yet research evidence around these factors is mixed. For instance, while some find higher rates of hate crime in disadvantaged neighbourhoods that also share similarly high rates of non‐hate crime, others find that hate crime has unique drivers from non‐hate offences and is most prominent in affluent communities (Lyons 2008). Further still, findings vary depending on the victim group targeted, data source utilised (official crime reports vs. victimisation surveys) and the broader cultural context of the study. Synthesising the available literature is thus critical for developing a clearer understanding of the place‐based factors associated with hate crime to guide prevention strategies.

### 1.2 Defining Hate Crime

The positioning and definition of hate crimes as a unique legal category is relatively new in the literature, having only emerged in the 1970s despite intergroup victimisation being a ‘historically familiar’ conduct (Jenness 2007, 142). As crime is a socially‐defined construct and differs by historical and cultural context, social norms and political interests, hate crime definitions developed for the purposes of jurisdictions' hate crime laws vary within and between countries (Boeckmann and Turpin‐Petrosino 2002; Perry 2002). As such, there is some debate in the academic literature about terminology and definitions, with scholars criticising definitions for being too ambiguous (Vergani et al. 2022). These definitions may lack specificity that resulting in overlaps with definitions of terrorism, gang violence or political violence. There is also contention in the literature about defining the word ‘hate’ as a causal factor in this crime type. Many incidents included in the term ‘hate crime’ are a result of the nuances of prejudice, bigotry, hostilities and intolerances rather than ‘hate’ per se (Iganski 2008; McDevitt et al. 2010). This means that instances of hate crimes and hate incidents are often captured using terms such as anti‐Semitism, Islamophobia or homophobia.

Providing a clear definition for the purposes of this review is essential for consistency, comparability and clarity in reporting, ultimately enhancing utility for informing policy, practice and future research. For the purpose of this review, we have elected to employ the hate crime definition provided by the Organisation for the Security and Co‐operation in Europe (OSCE) which states that ‘a hate crime has taken place when a perpetrator has intentionally targeted an individual or property because of one or more identity traits or expressed hostility towards these identity traits during the crime’ (Organisation for Security and Co‐operation in Europe [OSCE] 2023). This highlights that a hate crime must comprise two elements – a criminal offence and a bias motivation. While there is contention over the groups that are included within definitions, our review will take a broad approach. We will include hate crimes that are motivated by race, religion, ethnicity, disability, gender identity and sexuality. These groups are the most common identities presented in the hate crime literature (for further discussion, see Perry 2002). Despite hate speech and harassment being inciteful and harmful in many circumstances, these are not universally criminal, and so we do not include them in our review.

#### 1.2.1 The Territorial Use of Space

Empirical evidence from the last thirty years shows that hate crimes are clustered within neighbourhoods (Benier et al. 2016; Benier 2017b; Drakulich et al. 2022; Lettieri et al. 2023) and occur near people's homes rather than in a more central location (Harlow 2005; Mason 2005; Roberts et al. 2013). In this period, scholars have sought to explain what features of a community inspire hate crimes to be used as a symbolic measure to repel certain residents. The territoriality of space is becoming of increased interest to hate crime scholars as they attempt to explain the ‘geography of hate’, or the way in which hate crime becomes a resource to exclude the out‐group (i.e., the minority group) from certain areas (Perry 2009, 67; see also Flint 2004). Territory is typically understood as a ‘device for simplifying and clarifying something else, such as political authority, cultural identity, individual autonomy or rights’ (Delaney 2005, 19). Territories construct forms of identity and difference, resulting in a stratified space that produces social conflict (Delaney 2005). Therefore, it is only when a boundary's meaning signifies or conveys social power that an enclosed space becomes a territory.

Territoriality is an essential component of the organisation and identification of human associations, such as cultures, societies and smaller collectives, and how they identify themselves in the social and material world. These processes are linked by cultural elements, such as linguistics, morality, ethics and religion (Raffestin 2012). Thus, territoriality shapes and is shaped by collective and individual identities. Delaney argues that ‘the most obvious effect of territory is to disempower others: to divide and conquer, to confine and immobilise, to exclude … to fragment and isolate’. Indeed, in the United States, the lived experience of race is not only in the identities that are created through labels, but also through the ‘spaces and places’ in which groups exist (Berry and Henderson 2002, 6). These locations have a shared understanding about the social significance of this space (Britton 2008), and boundaries symbolise both social and spatial divisions of difference (Bergesen and Herman 1998; Perry 2009; Webster 2003).

It is important to consider the role of space and territoriality when considering hate crime, especially within the neighbourhood. In all Western societies, there are spaces and places which are perceived as ‘belonging’ to different groups, despite these often being public open spaces. The occurrence of hate crime can be understood through the perceived need to defend these spaces against people who do not ‘authentically belong’ in such areas and may not align with the identity of others within the space (Kretsedemas 2013, 44). Thus, it could be said that the fear of losing control or power through the dilution of people who have the status to inhabit the territory may be a strong motivation for hate crime to exclude those who are perceived as a threat to the identity or economic position of the area.

### 1.3 Risk/Protective Factors

The systematic review aims to synthesise the research evidence to identify the conditions that create an environment conducive to hate crime. Understanding the risk and protective factors for hate crimes is crucial for developing effective prevention and intervention strategies. Using hate crime at the place‐based level as the outcome, we assess the associations of individual and/or place‐level antecedents and will include and report on all risk and protective factors identified in the primary research studies. We expect that these will primarily be socio‐demographic factors (such as the place's economic disadvantage, ethnic composition, residential turnover, and/or proportion of unemployed residents) or social processes (such as collective efficacy, social cohesion, attitudes to diversity). The review will also consider whether the risk and protective factors for hate crime vary according to moderating factors, such as the features of the crime, the victim, the place, and cultural contextual factors.

### 1.4 How the Risk/Protective Factors Might be Linked to the Outcome

Risk and protective factors are likely to demonstrate profound influences on the prevalence of hate crimes. Previous literature has debated the role of two theories in explaining hate crime centred within a geographic proximity. The first argues that hate crime, by definition, includes an underlying crime and that it therefore shares similar ecological drivers to any other crime (Gladfelter et al. 2015). This means that hate crime may be explained by social disorganisation theory, which positions crime to be a result of factors such as economic disadvantage, ethnic heterogeneity and residential turnover, creating a lack of effective social control mechanisms (Sampson et al. 1997). Social disorganisation theory further suggests that in areas where traditional social structures are weakened, subcultures or deviant groups may emerge. These subcultures can form around shared prejudices or ideologies that target specific groups based on race, ethnicity, religion or other characteristics. They may perpetuate and reinforce discriminatory attitudes, potentially leading to hate crimes as a means of expressing or enforcing these beliefs.

The competing perspective suggests that because a hate crime must have an overarching motivation of a bias against the targeted group, there may be more significant problems in the place, which have corresponding correlates of hate crime that are *distinct* from non‐hate offences. Resource competition theories propose that hate is most frequent when economic competition between ethnic groups increases, particularly in times of hardship where resources are scarce (Lyons 2007). In contrast, the defended neighbourhood theory suggests hate crime is most likely in economically and socially organised communities which are able to use resources to exclude racial outsiders, particularly when a sudden in‐migration threatens a neighbourhood which has long been a predominantly white area (Lyons 2007, 2008).

In addition to these theories of hate, there are further risk and protective factors that are likely to impact rates of hate crime. For example, environments permeated by prejudice and intolerance normalise discriminatory behaviour, further escalating these crimes. Political instability and economic hardship can exacerbate tensions, leading to scapegoating and heightened targeting of marginalised groups (Lyons 2007; Omelicheva and Webb 2021). Conversely, communities fortified by strong institutions promoting inclusivity, effective law enforcement that swiftly addresses hate crimes, and comprehensive education programs fostering empathy and tolerance tend to experience lower rates of hate crimes (Cogan 2002; Litvak et al. 2024). Clear policies and laws that protect vulnerable communities may also serve as deterrents (Jacobs and Potter 2000; King 2007). The interplay between these factors is intricate, influencing fluctuations in hate crime rates over time and highlighting the importance of comprehensive approaches that address both risk factors and protective measures to combat hate crimes effectively.

### 1.5 Why It Is Important to Do This Review

The harms of hate crime have been studied extensively over the past 50 years. Such harms are published widely in the academic literature and detail the impact on the immediate victim as well as their effects on the entire community and society as a whole (see, e.g., Dreher 2006; FitzGerald and Hale 1996; Human Rights and Equal Opportunity Commission 2004; Kelly et al. 1993; Noelle 2002). There is often a particular focus on the physical and psychological trauma, fear and intimidation, social division and polarisation, impact on marginalised groups and economic costs that stem from these events, and normalisation of prejudice more broadly (Benier 2017a; Perry 2015). Hate and prejudice continue to be an issue in society globally, with increasing waves of incidents reported around the pandemic, political campaigns and global conflicts (see, e.g., Croucher et al. 2020; Schaffner 2020).

Hate crime scholars have highlighted discrepancies in antecedents between states and countries (see, e.g., Benier et al. 2016). While some studies stress that hate crime, parallel to other types of crime, is most likely to occur in socially disadvantaged areas that report poverty, high population turnover and limited social connection, other studies argue that the underlying prejudice that motivates the ‘hate’ in the offence has a specific and distinct set of antecedents that provoke the attack. To our knowledge, there has been no systematic synthesis of findings to consider points of similarity and difference between different contexts.

Hate crime causes significant harm to victims and to those who share the victim's (perceived) identity, and fractures community bonds more broadly. This proposed review not only aims to advance the academic literature on hate crime, but also deliver a product that can be used to inform practical prevention strategies. Understanding the risk and protective factors for hate crime is fundamental to its prevention, and establishing whether these factors are universal or contextually dependent will allow for tailored resources to be developed in areas in which hate crimes are likely to occur. This review aims to produce a comprehensive synthesis of the spatial hate crime literature to provide policy‐makers and practitioners with an evidence‐based overview of the places that are likely to see the highest rates of hate crime victimisation. It aims to provide systematic evidence to aid the development of preventative measures to counter acts of hate, inform policy‐makers tasked with reducing hate crime, and help determine future research needs.

## 2 Objectives

The review aims to synthesise the current empirical evidence on the places factors associated with hate crime victimisation. The review will also consider whether the risk and protective factors for hate crime vary according to moderating factors, such as the features of the crime, the victim, the place, and cultural contextual factors.

The review aims to answer two key research questions:

1. What are the place‐based risk and protective factors associated with hate crime victimisation?
2. Does the relative strength of the different risk and protective factors vary according to moderating factors? Potential moderators include: levels of geographic aggregation, type of hate crime, victim group targeted, type of data source, macro‐cultural context and the theoretical framework used in the primary research.

## 3 Methods

### 3.1 Criteria for Considering Studies for This Review

#### 3.1.1 Types of Studies

To be eligible for inclusion, studies must have employed a sample that demonstrates variability in both (a) the value of the outcome and (b) the value of one or more risk or protective factors. That is, a study must include both people who have been victims of hate crime and those who have not (noting that studies do not need to have specifically recruited non‐victims – e.g., official crime rates have non‐victims as a denominator within the rate), *and* it must also include both people who have been exposed to the risk/protective factor and those who have not. This variation can be either measured at the individual level and then aggregated to the place level (e.g., percentage of residents who have a university degree) or it can be measured directly at the place level (e.g., population density).

We anticipate that most studies will use a cross‐sectional study design; however, we will also include longitudinal studies and case‐control studies, as long as this variation is present. At a minimum, all included studies must provide a quantitative assessment of the place‐level relationship between one or more measures of hate crime victimisation and one or more risk/protective factor(s).

We will extract effect size data from eligible studies, but will not analyse raw data. We will include published and unpublished studies in this review.

#### 3.1.2 Types of Participants

Our initial search focus was designed to be the neighbourhood. Given that there is no universal geographic definition for a neighbourhood and that the application of this term will vary across countries and research settings, we have expanded our focus to include all geographic areas and analyses. This means that we include studies that explore towns, cities, regions, neighbourhoods, communities, and so forth. We will be guided by the study authors, as we note that authors who research in the neighbourhood space typically devote some text to definitions of their geographic area of use. Although we recognise that studies use different geographic definitions, we provide the following examples for reference: in the United States, a neighbourhood is typically operationalised as census block groups or census tracts; in Australia, Statistical Area Level 1 or state suburbs; and in the United Kingdom, Lower Super Output Areas or Electoral Wards. Where possible, we will use the unit size as a moderating variable in our meta‐analyses.

#### 3.1.3 Risk/Protective Factors

We expect that the risk and protective factors that we identify will primarily be sociodemographic factors (such as economic disadvantage, ethnic composition, residential turnover and/or proportion of unemployed residents) or social processes (such as collective efficacy, social cohesion, attitudes to diversity).

We will differentiate between predictors and correlates in our review, and we expand on this distinction below. To be considered a reliable predictor, a factor must (at minimum) precede the outcome, which underscores the importance of longitudinal designs in identifying predictive factors (Farrington et al. 2012). However, many studies on hate crime victimisation employ cross‐sectional designs, where certain factors are reported retrospectively or exist before the incident (e.g., sex assigned at birth, ethnicity), while others are measured only after victimisation occurs (e.g., perceptions of tensions). We recognise the complexities introduced by the temporal ordering of measurement and that it can blur the distinction between causes of hate crime and its consequences, and thus we will distinguish between predictors and correlates.

The key distinction between predictors and correlates is whether temporal precedence, and therefore any plausible argument for causation, can be established. Our criteria for distinguishing between predictors and correlates build on the methodological approach used in Higginson et al. (2014).

*Predictors* are defined as either:

1. Time‐invariant factors (e.g., rural vs. urban place), or
2. Factors that were measured before the outcome occurs (i.e., before hate victimisation) (e.g., census data was collected in 2021, used to predict last‐year incidence of hate victimisation in 2024), or
3. Factors that were measured retrospectively to a time before the outcome occurring (e.g., participants were asked how often they interacted with their neighbours before the pandemic, used to predict last‐year incidence of hate victimisation in 2024).

*Correlates* are defined as either:

1. Factors that are measured in such a way that a clear temporal ordering cannot be established between the factor and the outcome (e.g., lifetime prevalence of offending, correlated with lifetime prevalence of hate crime victimisation), or
2. Factors that are measured at the same time that the outcome occurs (e.g., 2021 census data, correlated with 2021 police reports of hate crime victimisation).

We will exclude studies where the measure of hate victimisation clearly occurs before the measurement of the factor (e.g., studies that evaluate the impact of hate victimisation on community wellbeing).

To avoid making unwarranted causal attributions, we will synthesise the effect sizes for predictors and correlates separately.

#### 3.1.4 Types of Outcome Measures

Studies will be included in this review if they assess the following outcomes:

- Official records of hate crime incidents.
- Self‐reported hate crime victimisation.
- Self‐reported hate crime offending.
- Third‐party/agency‐reported hate crime incidents.

We will include hate crimes motivated by race, religion, ethnicity, disability, gender identity and sexual orientation. Any incident that is labelled as a hate crime by its study authors will be included, such as (but not limited to) physical assault, arson, graffiti and property damage. We will not include harassment or hate speech. Despite these being inciteful and harmful in many circumstances, these are not universally criminal and the inclusion of hate speech in these analyses would be contentious.

Studies that include both hate and non‐hate outcomes will be included in the review. However, only hate crime outcomes will be included in the meta‐analyses.

#### 3.1.5 Duration of Follow‐Up

This review focuses on the relationship between risk/protective factors and hate crime. As discussed under Section 3.1.1, studies may measure this relationship either at a single point in time (cross‐sectional studies) or at multiple points in time (longitudinal studies). Data may be collected prospectively or retrospectively.

We will exclude studies that measure the risk/protective factor only where it occurs after the hate crime incident. Other than this exclusion, we will not place constraints on the temporal relationship between the risk/protective factors and the experience of hate crime; however, we will use these temporal relationships to categorise the risk/protective factors as either predictors or correlates (see Section 3.1.3 for more detail).

#### 3.1.6 Types of Settings

We will not place any geographical limitation on our studies and will include studies from all countries. We will search in English, but will code all resulting studies in other languages with the use of Google Translate.

### 3.2 Search Methods for Identification of Studies

#### 3.2.1 Search Terms

To identify eligible studies, our search strategy will focus on four domains of search terms: Attribute; Behaviour; Geography and Methodology (see Table 1). We have drawn on the search terms used in Windisch et al. (2022) and Vergani et al. (2024), as well as conversations within our team, to create broad sets of search terms around the domains of Attribute and Behaviour, which together are highly sensitive to hate crimes. The Attribute domain captures the motivation or target of hate crime, and the Behaviour domain captures the actions associated with hate crime. The search terms in the Methodology domain are deliberately broad and our pilot searches demonstrate that including this domain increases specificity without sacrificing sensitivity.

**Table 1:** Search terms, by domain.

A. Attribute	B. Behaviour^a^	C. Methodology	D. Geography
hate OR prejudice OR ‘bias motivated’ OR ‘bias crime’ OR ‘identity‐based’ OR islamophobi* OR racis* OR racial* OR xenophobi* OR sinophobi* OR homophobi* OR transphobi* OR lesbophobi* OR biphobi* OR ableis* OR disablis* OR disableis* OR sexis* OR (anti NEAR/1 (foreigner OR migrant* OR immigra* OR refugee* OR ‘asylum seeker*’ OR Roma OR traveller* OR Gypsy OR gypsies OR ‘First Nation*’ OR Indigenous OR Māori OR Aboriginal* OR semiti* OR semiti* OR Jew* OR Amish OR Sikh OR Buddhis* OR Muslim* OR Islam* OR Christian* OR gay OR lesbian* OR bisex* OR transgender* OR LGBT* OR homosexual*))	crim* OR incident* OR abus* OR vilif* OR violen* OR rape* OR murder* OR micro‐aggression* OR microaggression* OR theft* OR robber* OR graffiti OR burglar* OR vandal* OR damage* OR arson* OR assault* OR homicide* OR lynch* OR offen* OR victim*	comparison* OR quantitative OR survey* OR poll* OR mixed‐method* OR ‘mixed method’ OR individual‐level OR group‐level OR control* OR evaluat* OR longitudinal OR random* OR analy* OR ‘risk factor*’ OR ‘protective factor*’ OR questionnaire* OR correlat* OR spatial OR geograph* OR statistic* OR record*	neighbo* OR district* OR area* OR local* OR communit* OR region* OR zone* OR precinct* OR ward* OR OR division* OR block* OR borough* OR council* OR postcode* OR ‘zip code*’ OR suburb* OR state* OR municipalit* OR spatial OR ‘census tract*’ OR city OR cities OR counties OR county

^a^ While we do not include vilification or micro‐aggressions in the review, as they are often not regarded as criminal offences, we do include them in the search terms. This is to ensure that we capture all possible studies due to variations in definitions and contexts, with studies with these minor infractions filtered out during screening.

As this review aims to locate primary studies that use the place (such as neighbourhood) as the unit of analysis, we have also included spatial area as a domain in our search strategy. While we initially tried searching without this category to obtain a broad number of studies, this resulted in an extremely large number of irrelevant results. We therefore included the geographical terms to further narrow down our search results to obtain a more specific set of documents that were more achievable to sort and screen.

To the extent that database functionality allows, each database will be searched using the search terms *within* each of the three domains (combined using the OR operator and proximity operators as appropriate), and these results will be combined *across* domains using the AND operator.

#### 3.2.2 Electronic Searches

We will search across the electronic databases shown in Table 2, and adapt the search syntax as necessary for each database's functionality.

**Table 2:** Search locations by host.

Platform	Database
EBSCOHost	LGBTQ+ Source Violence & Abuse Abstracts Criminal Justice Abstracts
Ovid	APA PsycArticles APA PsycBooks APA PsycInfo APA PsycExtra
ProQuest	Dissertation & Theses Global Criminal Justice Database Psychology Database Political Science PTSDPubs Social Science Database Social Services Abstracts Sociology Database Sociological Abstracts
Web of Science (via Clarivate)	Social Sciences Citation Index Arts and Humanities Citation Index Conference Proceedings Citation Index – Social Science & Humanities Book Citation Index – Social Sciences and Humanities Emerging Sources Citation Index
Scopus	Social Sciences
Informit	CINCH – Australian Criminology Database

Where possible, the search strategy will be executed in electronic databases across all indexed fields except full text (NOTF). If this is not possible, we will focus our search on the Title, Abstract, and Keyword fields as appropriate. We will limit the document type to avoid identifying documents that are very unlikely to contain quantitative research, such as book reviews, news articles, and so forth. As an example, Table 3 provides the full search strategy for Proquest Criminal Justice Database, with results limited by: Books, Conference Papers & Proceedings, Dissertations & Theses, Reports, Scholarly Journals.

**Table 3:** Proquest Criminal Justice Database search syntax.

S1	noft(hate OR prejudice OR ‘bias motivated’ OR ‘bias crime’ OR ‘identity‐based’ OR islamophobi* OR racis* OR racial* OR xenophobi* OR sinophobi* OR homophobi* OR transphobi* OR lesbophobi* OR biphobi* OR ableis* OR disablis* OR disableis* OR sexis* OR (anti NEAR/1 (foreigner OR migrant* OR immigra* OR refugee* OR ‘asylum seeker*’ OR Roma OR traveller* OR Gypsy OR gypsies OR ‘First Nation*’ OR Indigenous OR Māori OR Aboriginal* OR semiti* OR Jew* OR Amish OR Sikh OR Buddhis* OR Muslim* OR Islam* OR Christian* OR gay OR lesbian* OR bisex* OR transgender* OR LGBT* OR homosexual*)))))	*N* = 25,116
S2	Noft(crim* OR incident* OR abus* OR vilif* OR violen* OR rape* OR murder* OR micro‐aggression* OR microaggression* OR theft* OR robber* OR graffiti OR burglar* OR vandal* OR damage* OR arson* OR assault* OR homicide* OR lynch* OR offen* OR victim*)	*N* = 318,439
S3	noft(comparison* OR quantitative OR survey* OR poll* OR mixed‐method* OR ‘mixed method’ OR individual‐level OR group‐level OR control* OR evaluat* OR longitudinal OR random* OR analy* OR ‘risk factor*’ OR ‘protective factor*’ OR questionnaire* OR correlat* OR spatial OR geograph* OR statistic* OR record*)	*N* = 259,506
S4	noft(neighbo* OR district* OR area* OR local* OR communit* OR region* OR zone* OR precinct* OR ward* OR OR division* OR block* OR borough* OR council* OR postcode* OR ‘zip code*’ OR suburb* OR state* OR municipalit* OR spatial OR ‘census tract*’ OR city OR cities OR counties OR county)	*N* = 348,440
S5	S1 + S2 + S3 + S4	*N* = 4657

#### 3.2.3 Searching Other Resources

We will search grey literature sources in Table 4. We will also conduct reference harvesting of related systematic reviews and all studies that are screened as eligible for inclusion in the review. Finally, we will contact experts in the field to identify missing studies.

**Table 4:** Grey literature sources.

Source	Website
International Network for Hate Studies	https://internationalhatestudies.com/publications/
Hate Crime Research & Policy Institute, Florida State University	https://criminology.fsu.edu/center-for-criminology-and-public-policy-research/institutes/hate-crime-research-policy-institute
The Centre for Hate Studies, University of Leicester	https://le.ac.uk/hate-studies/research
Australian Institute of Criminology	www.aic.gov.au
Centre for Study and Reduction of Hate Crime Bias and Prejudice, Nottingham Trent University	https://www.internetjournalofcriminology.com/
Home Office UK	https://www.gov.uk/search/research-and-statistics
Government of Canada	https://www.canada.ca/en.html
Equality and Human Rights Commission, UK	https://www.equalityhumanrights.com/
European Union Agency for Fundamental Rights	https://fra.europa.eu/en/themes/hate-crime
Stop Hate UK	www.stophateuk.org
Public Safety Canada	https://www.publicsafety.gc.ca/index-en.aspx
Cochrane Library	https://www.cochranelibrary.com/search
Campbell Collaboration	https://www.campbellcollaboration.org/

### 3.3 Data Collection and Analysis

#### 3.3.1 Criteria for Determination of Independent Findings

We recognise the likelihood of dependencies in the data, and that these dependencies are likely to come from two major sources. First, there is the dependency that occurs when one study is conducted but is reported on in more than one document. These documents cannot be treated as independent studies, as they share the same data. After all documents are screened for eligibility, we will attempt to identify all related (but otherwise eligible) documents. Recognising that data may be reported differently across related documents, we will either select effect size data from one of the related documents (where it is sufficient), or the data may be drawn from a combination of related documents. These decisions will depend on the completeness of data reporting in each document and the risk of bias assessments. All decisions will be reported in the final review.

The second issue of dependency is that a document may report on more than one predictor or correlate and/or more than one measure of hate victimisation. If this occurs, we will allow a study to contribute multiple effect sizes to the review, but only one effect size to any meta‐analysis. In these cases, we will report all effect sizes for completeness, but calculate one mean effect size for inclusion in the meta‐analyses.

Related to this, we recognise that many of the included studies will be in the form of models that report both unadjusted and adjusted correlation coefficients. These nested effect sizes pose an additional challenge to meta‐analysis, as they present the questions of which effect size is ‘best’ and which effect size is most common. We will calculate both unadjusted and adjusted effect sizes and report both in the review. We will aim to find commonality between studies so that meta‐analyses can be performed wherever possible, and all decisions will be reported transparently. Where multiple options are obtained from a study, we will perform sensitivity analyses on the impact of different covariates on the final estimates.

#### 3.3.2 Selection of Studies

After conducting the searches specified above, the research team will download the bibliography details of relevant documents into DistillerSR for management. DistillerSR also provides access to the full text of available studies through HTML links. The selection of studies will then follow a two‐stage screening approach.

After quarantining duplicate entries, screening will be conducted on the titles and abstracts of identified studies. To ensure consistency of decision making, 5% of exclusions will undergo double screening to test for false negatives; otherwise, screenings will be conducted independently by the research team (without double‐screening). We also plan to engage AI screening with the DistillerSR software. We will manually screen the first 10,000 articles to create a training AI set, and then use AI to screen the remaining set. We will double‐screen 5% of the AI set for verification. We will also use the ‘Check for Screening Errors’ function in DistillerSR as an additional cross‐check for any potential false negative exclusions. Documents will be screened based on the following three (negatively framed) screening items:

1. Document does not address hate crime (Yes/No/Unsure).
2. Document does not have a geographical focus (Yes/No/Unsure).
3. Document does not use quantitative methods (Yes/No/Unsure).

If the answer to any of these three criteria is yes, the document is excluded from further assessment, and it will not proceed to full text screening. If the document cannot be excluded unambiguously, it will be advanced to the next stage for further assessment.

The full text will be retrieved for all documents that have passed through this first stage of screening. The second stage of screening then seeks to confirm eligibility based on the reading of the full text of the study, based on the screener's response to the following four (affirmatively framed) screening items:

1. Document assesses factors associated with hate crime (Yes/No/Unsure).
2. Document uses a geographical focus (Yes/No/Unsure).
3. Document uses quantitative methods (Yes/No/Unsure).
4. Document assesses factors associated with hate crime using a geographical focus and quantitative methods (Yes/No/Unsure).

A document will be included in the review if the answer to item 4 is yes. We will double‐screen 5% of the excluded studies, and if necessary, we will rescreen after further consensus. Ambiguities will be resolved by discussion within the research team, and if necessary, by contacting the document's author.

#### 3.3.3 Data Extraction and Management

Each study will be independently coded by two review authors, using the coding framework in Appendix A. This framework draws on previous Campbell Reviews, including Calderoni et al. (2019), Chambers et al. (2023), Higginson et al. (2018) and Wolfowicz et al. (2021). The form will be piloted before data extraction commences, and modifications will be noted in the subsequent systematic review report. Any disagreements between reviewers on data extraction will be resolved either through consensus or, if necessary, with the help of a third reviewer. For each document, we will note information about author/s, year, document type and country/region of focus. In line with MECCIR reporting standards, we will detail information on sample size, study design and quality, participants, outcome characteristics, effect size data, funding sources and conflicts of interest. During data extraction, we will verify the accuracy of all numeric data. If any information is missing from published reports, we will ask for further details from the study author/s via email.

#### 3.3.4 Assessment of Risk of Bias in Included Studies

We adapt the measures from Higginson et al. (2018) to assess risk of bias as part of the data extraction process, using the following items:

1. Study population description. Does the document describe the source population in replicable detail? (Yes, No, Unclear).
2. Study population criteria: Does the document list all inclusion and exclusion criteria for participation? (Yes, No, Unclear).
3. Outcome descriptor: Were the hate crime measurement criteria described in replicable detail? (Yes, No, Unclear).
4. Predictor description: Were all predictors described in replicable detail? (Yes, No, Unclear).
5. Predictor validity: Were all measures of the predictors based on a validated measure? (Yes, No, Unclear).
6. Predictor timing: Were all predictors either measured before victimisation or measured retrospectively to a time before victimisation? (Yes, No, Unclear).
7. Selective predictor reporting: was the study free from predictor reporting bias? (Yes, No, Unclear).
8. Selective analysis reporting: was the study free from analysis reporting bias? (Yes, No, Unclear).

We also added an additional item: Is the non‐exposed group (control) reasonably comparable to the exposed (hate victims) group? (Yes, No, Unclear) to assess the effectiveness of the comparison group.

We will not calculate an overall rating of risk of bias for each study, rather will present the results of each domain for each study in a ‘traffic light’ format (see De Vibe et al. 2012). Risk of bias will be assessed by two reviewers, with discrepancies resolved by discussion and/or a third reviewer. We will not exclude studies based on their risk of bias assessment; instead, we will perform sensitivity analyses to examine the degree to which risk of bias affects the overall summary effect size.

#### 3.3.5 Measures of Association

Following Calderoni et al. (2019), we anticipate that eligible studies will allow the calculation of effect sizes across three main categories: mean differences, odds ratios, and correlation or regression coefficients.

We will calculate Hedges' *g* for studies that provide mean differences between groups on a continuous measure, and calculate the log odds ratio for studies that provide binary predictors and outcomes. We will calculate Fisher's *z* for raw, unadjusted correlations between predictors and outcomes. Depending on the data, we will calculate either the standardised mean difference (Cohen's *d*) or the semi‐partial correlation coefficient (Aloe and Thompson 2013) for studies that use multiple regression models, depending on the form of the data. If such studies also include zero‐order correlations, we will calculate Fisher's *z* to compare the effect of using bivariate correlations and partial regression coefficients in our sensitivity analyses. Information on the covariates used in such studies will also be coded, and if data permits, we will perform sensitivity analyses on the impact of different covariates on the final estimates. As discussed above, we anticipate that many of the included studies will be in the form of regression analyses, and that multiple regression poses distinct challenges to meta‐analysis.

Effect sizes will be converted where appropriate to ensure a common metric for each meta‐analysis. Ideally, we will use the log odds ratio in the meta‐analyses, in which case the results will be presented as the odds ratio for ease of interpretation. We recognise that these conversions are not always ideal, but are preferable to separate meta‐analyses by effect size (Borenstein 2009).

#### 3.3.6 Unit of Analysis Issues

We expect that most of the studies that are eligible will include census data variables that predict a rate or a count of victimisation within a fixed area. It is also likely to have studies that include individual/household nested data in places that predict victimisation. To our knowledge, we are unlikely to find studies that include multiple outcome measurement time‐points, as studies of this nature are cross‐sectional rather than longitudinal analyses.

#### 3.3.7 Dealing With Missing Data

Ideally, we will calculate effect sizes from the statistics reported in the study (e.g., *t*, *F*, *p*, *z*). We will calculate effect sizes from tabulated data, where necessary, but will not calculate effect sizes from raw data. Where effect sizes cannot be calculated, we will contact study authors to seek further details. Studies that cannot be included in the meta‐analyses because effect sizes cannot be calculated will be reported and included for discussion in the final review.

#### 3.3.8 Data Synthesis

Assuming that the systematic search provides at least two eligible studies that report on the same predictor‐outcome relationship, we will calculate the weighted mean estimate using a random‐effects meta‐analysis with inverse variance weighting for each predictor‐outcome association. Results will be presented in forest plots with 95% confidence intervals.

Where meta‐analysis is not possible, we will plot the effect sizes and 95% confidence intervals in a forest plot without a weighted mean estimate.

Whilst we hope to be able to calculate effect sizes for all eligible studies, we recognise that missing data may preclude this. If effect sizes are not able to be calculated, the study will still be included in the report, and the author's interpretation of their study results will be discussed.

#### 3.3.9 Assessment of Heterogeneity

We follow Borenstein (2009) in estimating the effect size heterogeneity using *I* ^2^, *τ* ^2^ and *Q*. These will be reported in the review alongside the relevant meta‐analyses.

#### 3.3.10 Subgroup Analysis and Investigation of Heterogeneity

Where sufficient data are available, we will code an a priori list of moderators for each study and conduct subgroup analyses to assess their impact on the effect size. Moderators will include country, covariates (number/type), type of hate, timeframe, geographic size (such as census tract, neighbourhood or county as examples) and study design. We will differentiate between a priori and post hoc moderator analysis in the final review.

#### 3.3.11 Assessment of Reporting Biases

We will conduct a sensitivity analysis around the risk of bias by comparing results from published and unpublished literature. We will also model publication bias and small study effects using funnel plots and trim‐and‐fill analysis as suggested in Rothstein et al. (2005).

#### 3.3.12 Sensitivity Analysis

We will perform subgroup analyses to evaluate how study quality and design influence outcomes. We anticipate conducting sensitivity analyses on the following variables: risk of bias, publication status, publication year, controlled versus raw correlation coefficients, and unit of analysis.

#### 3.3.13 Treatment of Qualitative Research

We will not include qualitative research.

#### 3.3.14 Summary of Findings and Assessment of the Certainty of the Evidence

At the conclusion of the final review, we will present a discussion of the key findings and an assessment of the overall quality of the evidence based on our reported results.

## Supporting information

Appendix.
