# Supplementary material for: PROTOCOL: Correlates and Antecedents of Hate Crime: A Systematic Review of Place‐Level Risk and Protective Factors
Source: Campbell Syst Rev. 2025 Jul 7;21(3):e70048. doi: 10.1002/cl2.70048 (PMC12230862; doi:10.1002/cl2.70048)
Supplement: Supplementary file 1 — Appendix. [file CL2-21-e70048-s001.docx]

**Appendix A: Document coding protocol**

Reference information

1. Document ID
2. Study author/s
3. Study title
4. Publication year
5. Full APA reference
6. Reference type:

a. Book/Book chapter

b. Journal article (peer reviewed)

c. Dissertation or thesis

d. Research report

e. Conference paper

f. Other (specify)_____________________

1. Coder’s name; date coded (Autofilled in Distiller)
2. Country studied_________________________
3. Document language ___________________________
4. Date of data collection
   1. Start: ____________
   2. Finish: ____________
   3. Dates not provided
5. Source of funding for study (relative to study location)
   1. Government
   2. Foreign government
   3. Local university/research body
   4. Foreign university/research body
   5. Other _________________
   6. No data provided
6. Funding conflict of interest (yes / no / insufficient information)
7. Author conflict of interest (yes / no / insufficient information)

Methodology

1. Sample size N ___________
   1. Exposure (hate victims) group n ________
   2. Non-exposed (may be non-victim, or non-hate victim etc) group n __________
2. Sample age (for example mean or range) _________________
3. Sample sex
   1. Male %
   2. Female %
4. Ethnic background (eg general population, or a specific identity of victims) _______
5. Recruitment strategy (eg random, representative, snowball etc) _____________
6. Settings where participants were recruited (eg schools, community hub etc) _________
7. Response rate ______
8. Geographic area (as defined by authors):
   1. Census tract / block
   2. Community / neighbourhood / suburb
   3. Local Government Area
   4. City
   5. Region / County
   6. Other ________________
   7. How does the author describe the exact geographic area? ______________
9. Type of study
   1. Longitudinal
   2. Cross-sectional
   3. Case control
   4. Other ______________
10. Data source of hate crime
    1. Administrative data (eg police)
    2. Self-report (eg survey)
    3. Self-reported to external agency/register
    4. Other _______
11. Type of crime
    1. All offence types
    2. Property damage
    3. Violent offences
    4. Other __________
12. Terms used by author to describe motivation (eg hate, prejudice, bias)_________
13. How many risk factors are reported in the study? __________
14. Evidence of ethical issues (yes / no)

Risk of bias

1. Does the document describe the source population in replicable detail?
   1. Yes
   2. No
   3. Unclear
2. Does the document list all inclusion and exclusion criteria for participation?
   1. Yes
   2. No
   3. Unclear
3. Is the non-exposed group (control) reasonably comparable to the exposed (hate victims) group?
   1. Yes
   2. No
   3. Unclear
4. Were the hate crime measurement criteria described in replicable detail?
   1. Yes
   2. No
   3. Unclear
5. Predictor description: Were all predictors described in replicable detail?
   1. Yes
   2. No
   3. Unclear
6. Were all measures of the predictors based on a validated measure?
   1. Yes
   2. No
   3. Unclear
7. Were all predictors either measured before victimisation or measured retrospectively to a time prior to victimisation?
   1. Yes
   2. No
   3. Unclear
8. Was the study free from predictor reporting bias?
   1. Yes
   2. No
   3. Unclear
9. Was the study free from analysis reporting bias?
   1. Yes
   2. No
   3. Unclear

Risk factors (nested coding for each factor)

1. Risk factor ________
2. Operational definition ________
3. Source of risk factor measure
   1. Self-reported
   2. Official data
   3. Other ______
4. Risk factor measured retrospectively (yes / no / unsure)
5. Is the risk factor time invariant (yes / no / unsure)
6. Was the standardised effect size reported (yes / no)
7. If yes, page number of effect size ______
   1. Effect size measure (r, b, B(exp), OR, other ______)
   2. Effect size ________
   3. Standard error of effect size _____________
   4. CI of effect size __________________
8. If no, is there data to calculate an effect size (yes / no)
   1. If yes, type of data effect size can be calculated from:

a. Means and standard deviations

b. Frequencies or proportions (dichotomous)

c. Frequencies or proportions (polychotomous)

d. Unadjusted correlation coefficient

e. Multiple regression coefficients (unstandardized)

f. Multiple regression coefficients (standardized)

g. Odds ratio(s)

h. t-value or F-value

i. Chi-square

j. Other (specify) ___________

Means and standard deviations:

- 1. Hate victim mean. ______
  2. Comparison group mean. ______
  3. Hate victim standard deviation. _____
  4. Comparison group standard deviation. _____

Proportions or frequencies

- 1. n of hate victim group with the predictor. _____
  2. n of comparison group with the predictor. _____
  3. Proportion of hate victim group with the predictor. _____
  4. Proportion of comparison group with the predictor. _____

Regression coefficients and correlations

- 1. Unadjusted correlation coefficient___________
  2. Standardized regression coefficient______
  3. Unstandardized regression coefficient______
  4. Standard deviation of predictor _______
  5. Control variables _________________________________

Significance Tests

- 1. t-value _____
  2. F-value _____
  3. Chi-square value _____

1. Anything else to add? (free text)
2. Calculated effect size
   1. Effect size ______
   2. Standard error of effect size _____
3. Page number of effect size information
4. What did the authors conclude about the relationship between the factor and hate crime?
   1. Factor increased hate crime
   2. Factor decreased hate crime
   3. Factor had no influence on hate crime
   4. Unclear / no conclusion stated by authors
